# Progression of myocardial fibrosis in hypertrophic cardiomyopathy: mechanisms and clinical implications

**DOI:** 10.1093/ehjci/jey135

**Published:** 2018-10-24

**Authors:** Betty Raman, Rina Ariga, Marco Spartera, Sanjay Sivalokanathan, Kenneth Chan, Sairia Dass, Steffen E Petersen, Matthew J Daniels, Jane Francis, Robert Smillie, Adam J Lewandowski, Eric O Ohuma, Christopher Rodgers, Christopher M Kramer, Masliza Mahmod, Hugh Watkins, Stefan Neubauer

**Affiliations:** 1 Division of Cardiovascular Medicine, Radcliffe Department of Medicine, University of Oxford, Headley Way, Oxford, UK; 2 William Harvey Research Institute, NIHR Barts Biomedical Research Centre, Queen Mary University of London, London, UK; 3 Barts Heart Centre, St Bartholomew’s hospital, Barts Health NHS Trust, West Smithfield, London, UK; 4 Nuffield Department of Orthopaedics, Rheumatology and Musculoskeletal Sciences, Centre for Statistics in Medicine, University of Oxford, Old Road Campus, Oxford, UK; 5 Centre for Tropical Medicine and Global Health, Nuffield Department of Medicine, University of Oxford, Old Road Campus, Oxford, UK; 6 Department of Clinical Neurosciences, Wolfson Brain Imaging Centre, University of Cambridge, Cambridge, UK; 7 Cardiology and Radiology, University of Virginia Health System, Charlottesville, VA, USA

**Keywords:** hypertrophic cardiomyopathy, fibrosis progression, microvascular dysfunction, clinical outcomes, myocardial energetics, late gadolinium enhancement

## Abstract

**Aims:**

Myocardial fibrosis as detected by late gadolinium enhancement (LGE) on cardiac magnetic resonance (CMR) is a powerful prognostic marker in hypertrophic cardiomyopathy (HCM) and may be progressive. The precise mechanisms underlying fibrosis progression are unclear. We sought to assess the extent of LGE progression in HCM and explore potential causal mechanisms and clinical implications.

**Methods and results:**

Seventy-two HCM patients had two CMR (CMR1-CMR2) at an interval of 5.7 ± 2.8 years with annual clinical follow-up for 6.3 ± 3.6 years from CMR1. A combined endpoint of heart failure progression, cardiac hospitalization, and new onset ventricular tachycardia was assessed. Cine and LGE imaging were performed to assess left ventricular (LV) mass, function, and fibrosis on serial CMR. Stress perfusion imaging and cardiac energetics were undertaken in 38 patients on baseline CMR (CMR1). LGE mass increased from median 4.98 g [interquartile range (IQR) 0.97–13.48 g] to 6.30 g (IQR 1.38–17.51 g) from CMR1 to CMR2. Substantial LGE progression (ΔLGE ≥ 4.75 g) occurred in 26% of patients. LGE increment was significantly higher in those with impaired myocardial perfusion reserve (<MPRI 1.40) and energetics (phosphocreatine/adenosine triphosphate <1.44) on baseline CMR (*P* ≤ 0.01 for both). Substantial LGE progression was associated with LV thinning, increased cavity size and reduced systolic function, and conferred a five-fold increased risk of subsequent clinical events (hazard ratio 5.04, 95% confidence interval 1.85–13.79; *P* = 0.002).

**Conclusion:**

Myocardial fibrosis is progressive in some HCM patients. Impaired energetics and perfusion abnormalities are possible mechanistic drivers of the fibrotic process. Fibrosis progression is associated with adverse cardiac remodelling and predicts an increased risk of subsequent clinical events in HCM.

## Introduction

Sudden cardiac death (SCD) and advanced heart failure are recognized complications of hypertrophic cardiomyopathy (HCM).^1^ Myocardial fibrosis is an important substrate for both life-threatening arrhythmia and adverse cardiac remodelling^2^ in HCM. Histopathological studies confirm a high burden of fibrosis in both young adults^3^ who suffered a SCD and older patients with end-stage heart failure and HCM.^4^

Cardiovascular magnetic resonance (CMR) permits the *in vivo* assessment of myocardial fibrosis using late gadolinium enhancement (LGE) imaging.^4–6^ The presence and extent of LGE are emerging predictors of cardiovascular morbidity and mortality in HCM and not limited to adults.^7^^,^^8^ Recently, a significant proportion of children and adolescents with HCM were found to have LGE with evidence of progression on serial imaging.^9^ Longitudinal studies examining the rate of LGE progression at longer intervals are sparse^9–11^ with a lack of studies examining the clinical relevance of fibrosis progression. Mechanisms driving fibrosis progression in HCM are also incompletely understood.^9^

The myocardium in HCM exhibits characteristic abnormalities in substrate metabolism and vascular remodelling.^12–14^ For example, reduced phosphocreatine to adenosine triphosphate concentration ratio (PCr/ATP) on phosphorus magnetic resonance spectroscopy (^31^P-MRS) is a marker of abnormal energy utilization in HCM and may play a critical role in its pathophysiology.^13^^,^^15^ Similarly, microvascular dysfunction may trigger fibrosis in HCM, promoting contractile dysfunction.^4^^,^^14^ Dissecting the pathophysiological factors that cause fibrosis, rather than merely associate, remains a challenge.

Here, we sought to characterize the natural history of myocardial fibrosis in HCM and explore potential underlying mechanisms. We assessed whether the extent of LGE progression can serve as a predictor of clinical events to guide future management.

## Methods

### Population

This is a retrospective analysis of data from an observational study approved by local ethics committee (reference: 07/Q1607/66, 12/LO/1979). All patients with HCM enrolled in this study were recruited from the University of Oxford Inherited Cardiac Conditions Clinic and all were invited to have a repeat CMR as a part of the study. Genetic screening was undertaken for 13 HCM genes and mitochondrial mutations (see Supplementary data online). Diagnosis of HCM was based on the presence of unexplained left ventricular hypertrophy (LVH) (maximum left ventricular wall thickness, LVWT ≥ 15 mm) or the presence of a pathogenic HCM-causing sarcomeric mutation (genotype positive phenotype negative, G+P− patients included).

**Figure 1:**
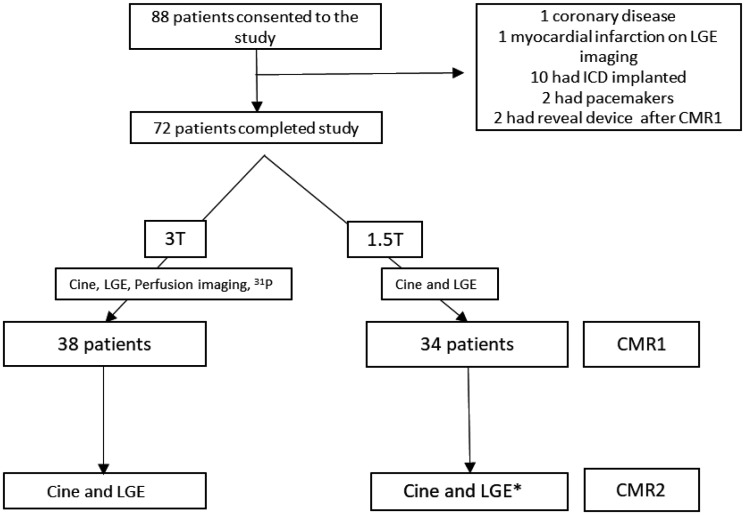
A flowchart of hypertrophic cardiomyopathy (HCM) patients through the study. CMR, cardiovascular magnetic resonance imaging; ICD, implantable cardioverter defibrillator; LGE, late gadolinium imaging; ^31^P, Phosphorus-31 spectroscopy; T, Tesla. *CMR2 was at 1.5T or 3T (see Supplementary data online).

Patients with known coronary artery disease, aortic stenosis, amyloidosis, or contraindications to CMR were excluded. A total of 88 patients were included in the study. Of them, 16 were excluded after CMR1. Ten had ICD’s implanted, two had pacemakers, two had reveal devices implanted, one had LGE in a myocardial infarction pattern and one had significant coronary disease on coronary angiography leaving 72 patients with two CMR scans (CMR1-CMR2) (*Figure 1*).

### CMR protocol

All 72 patients had serial CMR including cine and LGE assessment at 1.5T or 3T (see Supplementary data online) at an interval of 5.7 ± 2.8 years (*Figure 1*). Sixteen patients had follow-up CMR at different field strengths. Thirty-eight patients also had first pass perfusion imaging and myocardial energetics assessment at baseline (CMR1) (*Figure 1*), all 38 had serial CMR at the same field strength (3T). Cine was undertaken using a series of single breath-hold balanced steady-state free precession images for estimation of cardiac volumes and function as previously described.^16^

LGE imaging was acquired in multiple short-axis slices to match cine views and long-axis planes approximately 8–10 min after intravenous administration of the gadolinium-based contrast agent (GBCA) (total dose 0.15 mmol/kg) for all scans (see Supplementary data online). The inversion time was adjusted for optimal nulling of remote normal myocardium.^17^ For all CMR scans before 2012, Gadodiamide (Omniscan, Nycomed Amersham, UK) was used as contrast agent. Due to the emerging safety concerns of Gadodiamide (Omniscan), in particular the associated risk of nephrogenic systemic fibrosis, Gadobuterol (Gadovist^®^, Bayer Inc., Toronto, ON, Canada) was used as contrast agent at 1.5T and Gadoterate meglumine (Dotarem, Guerbet LLC, France) at 3T for all scans from 2012.^18–20^

Perfusion imaging was undertaken (before LGE imaging) at 3T on CMR1 for 38 patients using a T1-weighted gradient echo sequence with saturation recovery magnetization preparation. Adenosine was used as pharmacological stress at a rate of 140 µg/kg/min and up-titrated by haemodynamic response. Three short-axis slices (base, mid, and apex) were acquired. 0.03 mmol/kg of GBCA was injected at 6 mL/s during stress followed by a saline flush 12 mL at 6 mL/s and the same dose for rest acquisition. Three patients were excluded [failure of contrast injection (*n* = 2) or intolerance to adenosine (*n* = 1)]. The remaining patients were adequately stressed as evidenced by the presence of appropriate haemodynamic response and splenic switch off.^16^

^31^P MRS was performed on baseline CMR (3T) to measure myocardial energetics for 38 patients. Subjects were placed prone with their hearts over the centre of the coil as previously described.^16^ The PCr/ATP ratio from a mid-ventricular septal voxel in a position matching mid ventricular perfusion slice was obtained (see Supplementary data online).

### CMR image analysis

Commercially available software (Circle Cardiovascular Imaging Inc., Calgary, Canada) was used to analyse left ventricular (LV) volumes, mass, ejection fraction, peak systolic 2D global longitudinal, circumferential and radial strain as previously reported.^21^ The assessment of LV indices and LGE mass were undertaken by two observers blinded to the clinical information (M.S. and S.S.). Quantitative analysis of LGE was undertaken by setting a signal intensity threshold at five standard deviations (5 SD) above the mean intensity of a reference region of interest placed in a remote area of myocardium with no visual evidence of enhancement.^6^^,^^22^ A binary visual score (1 = progression; 0 = no progression) was also provided by an experienced (>5 years) CMR clinician (M.M.) to assess level of agreement between semiquantitative assessment and observed changes seen by an expert clinician (see Supplementary data online).

For perfusion analysis, signal intensity curves were generated to measure myocardial perfusion reserve index (MPRI) as previously described.^17^

Post-processing of ^31^P-MRS data was performed using the OXSA toolbox (see Supplementary data online).

### Clinical follow-up

Clinical follow-up was performed annually for a period of 6.3 ± 3.6 years from CMR1. In cases of a suspected event, all medical records were obtained and reviewed by two observers (B.R. and M.M.) blinded to the CMR data.

Major risk factors for SCD included traditional risk factors (see Supplementary data online). Additionally, the European Society of Cardiology (ESC) risk calculator was used to estimate 5-year SCD risk for all patients.^23^

Given the low event rate in this selected population undergoing serial CMR, we used a composite clinical endpoint of: heart failure progression defined as a progressive increase in New York Heart Association (NYHA) class necessitating optimisation of medical therapy, new onset non-sustained ventricular tachycardia (≥3 heart beats, ≥120 bpm), and hospitalization from cardiac cause (arrhythmia or heart failure). A change in NYHA class or medical therapy due to intolerance to medications did not constitute a clinical event in this study.

### Statistical analysis

Statistical analyses were undertaken using IBM SPSS Statistics 23.0 (IBM Corp., Armonk, NY, USA), STATA/SE 15.0 (Stata Corp, College Station, TX, USA), and GraphPad Prism 7.0 (GraphPad Software, San Diego, CA, USA). Normality of data was assessed by visually inspecting the plots. Mean (with standard deviation, SD) and median (with interquartile range, IQR or confidence intervals, CI for median differences) were computed as appropriate. Paired *t*-test and Mann–Whitney tests were used for normally distributed and non-Gaussian data respectively. The *χ*^2^ and Fisher’s exact tests were used to compare proportions. Given the current lack of a generally accepted cut off for ‘significant’ LGE progression (ΔLGE), a receiver operator curve was used to estimate the optimal ΔLGE threshold (Youden index) predictive of clinical events, which was an increment of 4.75 g (see Supplementary data online, *Figure S1*). Univariate and multivariable binary logistic regression were used to assess predictors of ΔLGE ≥ 4.75 g (binary variable).^24^ LGE mass at CMR1 was treated as a continuous variable. Kaplan–Meier curves were computed to visualize the cumulative patients event rates. A multivariable Cox proportional hazard model was used to analyse independent associations with clinical outcomes. The covariates included were variables known to be potential confounders and were adjusted for in the model. All tests were two-tailed and *P*-values <0.05 (after Bonferroni correction) were considered significant.

## Results

### Study population

The final population consisted of 72 patients with paired CMR data. *Table 1* lists their background characteristics at baseline CMR (CMR1) and second CMR (CMR2).

**Table 1:** Baseline characteristics of patients with HCM at CMR1 and CMR2

	CMR 1 (*n* = 72)	CMR 2 (*n* = 72)	*P*-value
Age (years)	45 ± 12	51 ± 12	<0.001
Male, % (*n*)	68 (49)	68 (49)	1.00
Body mass index (kg/m^2^)	27 ± 5	27 ± 5	0.08
Hypertension, % (*n*)	10 (7)	11 (8)	1.00
Diabetes, % (*n*)	3 (2)	6 (4)	0.68
Smoker, % (*n*)	7 (5)	6 (4)	1.00
Atrial fibrillation, % (*n*)	7 (5)	10 (7)	0.76
SCD risk			
Family history of SCD, % (*n*)	26 (19)	26 (19)	1.00
Unexplained syncope, % (*n*)	4 (3)	4 (3)	1.00
NSVT on Holter monitor, % (*n*)	10 (7)	24 (17)	0.04
Abnormal exercise BP response, % (*n*)	1 (1)	3 (2)	1.00
Maximum LV wall thickness ≥30 mm, % (*n*)	4 (3)	4 (3)	1.00
LV outflow tract gradient, % (*n*)	15 (11)	15 (11)	1.00
NYHA Class I, II, III, IV, % (*n*)	82, 14, 4, 0 (59, 10, 3, 0)	67, 25, 8, 0 (48, 18, 6, 0)	0.11
ESC risk score	2.01 ± 0.86	2.31 ± 1.44	0.01
SCD risk factors (0/1/2/3 risk factors), % (*n*)	61, 33, 6, 0 (44, 24, 4, 0)	47, 46, 6, 1 (34, 33, 4, 1)	0.29
Medications			
β-Blockers, % (*n*)	50 (36)	65 (47)	0.06
Calcium channel blockers, % (*n*)	8 (6)	17 (12)	0.27
Disopyramide, % (*n*)	6 (4)	15 (11)	0.09
ACEI/ARB, % (*n*)	14 (10)	14 (10)	1.00
Diuretics, % (*n*)	6 (4)	7 (5)	1.00
Aspirin, % (*n*)	25 (18)	47 (34)	0.006
Warfarin, % (*n*)	6 (4)	11 (8)	0.36
CMR findings			
LVEF (%)	67 ± 6	67 ± 7	0.44
LVEDV (mL)	152 ± 30	155 ± 32	0.12
LVEDV index (mL/m^2^)	79 ± 14	79 ± 14	0.43
LVESV (mL)	51 ± 15	51 ± 18	0.67
LA diameter (in LVOT/three-chamber view)	37 ± 6	37 ± 7	0.37
Stroke volume (mL)	101 ± 19	104 ± 20	0.11
LV mass (g)	146 ± 52	151 ± 52	0.02
LV mass index (g/m^2^)	75 ± 25	76 ± 27	0.15
Max LVWT(mm)	19 ± 6	19 ± 5	0.79
Presence of LGE, % (*n*)	75 (54)	82 (59)	0.31

Data are represented as mean ± standard deviation.

ACEI, angiotensin converting enzyme inhibitor; ARB, angiotensin receptor blocker; BP, blood pressure; EDV, end-diastolic volume; EF, ejection fraction; ESC, European Society of Cardiology; ESV, end-systolic volume; HCM, hypertrophic cardiomyopathy; LA, left atrial; LGE, late gadolinium enhancement (5-SD); LV, Left ventricular; LVOT, left ventricular outflow tract; NSVT, non-sustained ventricular tachycardia; NYHA, New York Heart Association; SCD, sudden cardiac death; WT, wall thickness.

Mean age of patients at CMR1 was 45 ± 12 years and 68% were male. At CMR1, the majority (94%) had one or no SCD risk factors, and four (6%) had two or more SCD risk factors (*Table 1*). The mean 5 year estimated risk of SCD on ESC risk calculator was low at 2.01 ± 0.86%.

By CMR2, patients were more likely to receive aspirin. The ESC 5-year estimated risk of SCD was also slightly higher (2.31 ± 1.44%, *P* = 0.01) at CMR2 (*Table 1*). Other baseline characteristics did not vary significantly.

### Influence of field strengths and contrast agents on LGE progression

In this observational study, 16/72 patients had follow-up CMR at different field strengths. Despite this, there was no association between changing field strength and fibrosis progression (*β* = −0.08, *P* = 0.64). We further assessed if the use of a specific combination of GBCA was associated with LGE progression. On univariate analysis, there was no association between the varying GBCA combinations and LGE progression (*β* −0.73, *P* = 0.10).

### Left ventricular volumes, function, mass, and LGE from CMR1 to CMR2

The mean LV end-diastolic volume (LVEDV), LV ejection fraction (LVEF), and mass at CMR1 were 152 ± 30 mL, 67 ± 6%, and 146 ± 52 g respectively. LVEDV and LVEF did not differ between CMR1 and CMR2 (*Table 1*). In contrast, a significant increase in LV mass between scans was detected (146 ± 52 g vs. 151 ± 52 g, *P* = 0.02) (*Table 1*). Modest reductions in both peak LV global circumferential (GCS) and longitudinal (GLS) strain were also seen from CMR1 to CMR2 (GCS −18 ± 3% vs. −17 ± 4%, GLS −17 ± 3% vs. −16 ± 3%; *P* < 0.05 for both).

**Figure 2:**
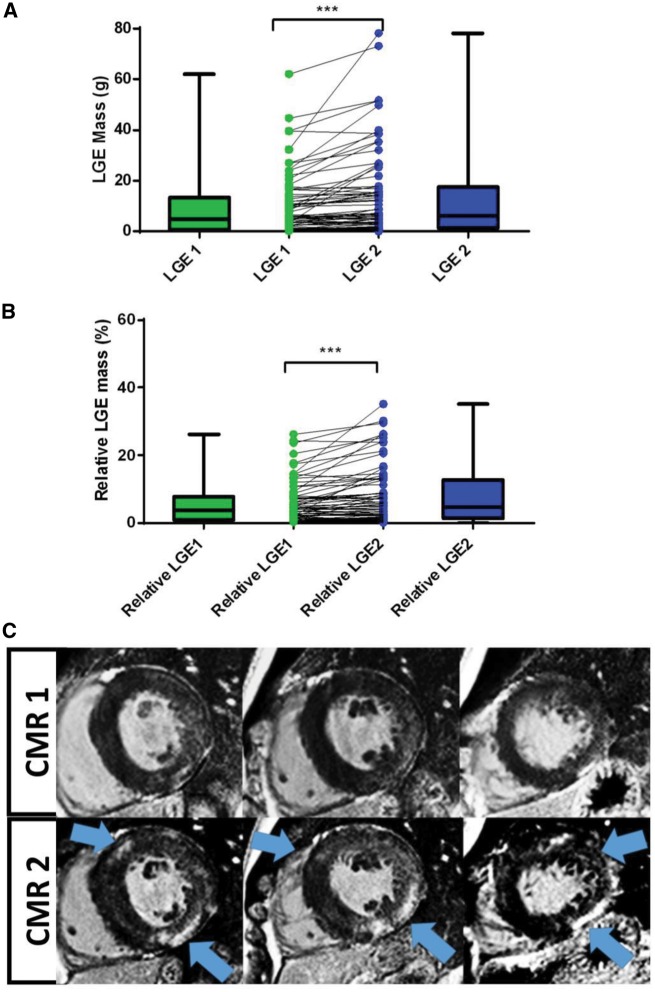
Comparison of (*A*) LGE mass and (*B*) relative LGE mass from CMR1 to CMR2 (***P < 0.0001, error bars represent SD). (*C*) a representative case of fibrosis progression in HCM (blue arrows indicate new regions of fibrosis).

LGE was present in 75% of HCM patients at CMR1, increasing to 82% at CMR2 (*Table 1*). LGE mass progressed from a median 4.98 g (IQR 0.97–13.48 g) on CMR1 to 6.30 g (IQR 1.38–17.51 g) on CMR2 (*Figure 2A*). As a relative proportion of LV mass, the median increment was 0.74% (95% CI 0.25–1.27%, *P* < 0.0001) from CMR1 to CMR2 (*Figure 2B*). LGE increment ≥4.75 g was seen in 26% (*n* = 19) of patients.

On univariate analysis, maximum LVWT, LV mass, and LGE mass at CMR1 were significant predictors of ΔLGE ≥ 4.75 g (*Table 2*). On multivariable analysis, LGE mass at CMR1 remained the only predictor of ΔLGE ≥ 4.75 g (*Table 2*).

**Table 2:** Univariate and multivariable predictors of LGE progression (ΔLGE ≥ 4.75g)

	OR	95% CI	*P*-value
Univariate analysis			
Age at CMR1	1.01	0.97–1.06	0.70
Max LV wall thickness at CMR1	1.25	1.10–1.42	0.001
LV mass at CMR1	1.01	1.01–1.03	0.005
LGE mass at CMR1	1.13	1.06–1.21	<0.001
LVEF at CMR1	0.94	0.86–1.02	0.13
Interval between CMR1-CMR2 (days)	1	1.00–1.01	0.50
Genotype^a^	1.93	0.55–6.73	0.30
Apical vs. non-apical hypertrophy	1.44	0.24–8.59	0.68
Baseline SCD risk (0 or ≥1)	0.47	0.15–1.48	0.19
Multivariable analysis			
Age at CMR1	1.01	0.95–1.07	0.76
Max LVWT at CMR1	1.14	0.96–1.34	0.14
LV mass at CMR1	0.99	0.99–1.01	0.94
LGE mass at CMR1	1.10	1.02–1.19	0.02

CI, confidence interval; CMR, cardiac magnetic resonance imaging; EF, ejection fraction; LGE, late gadolinium enhancement; LV, left ventricular; LVWT, left ventricular wall thickness; OR, odds ratio; SCD, sudden cardiac death.

a Sarcomeric and mitochondrial mutations vs. genotype negative.

### Relationship between LV wall thickness and LGE progression

**Figure 3:**
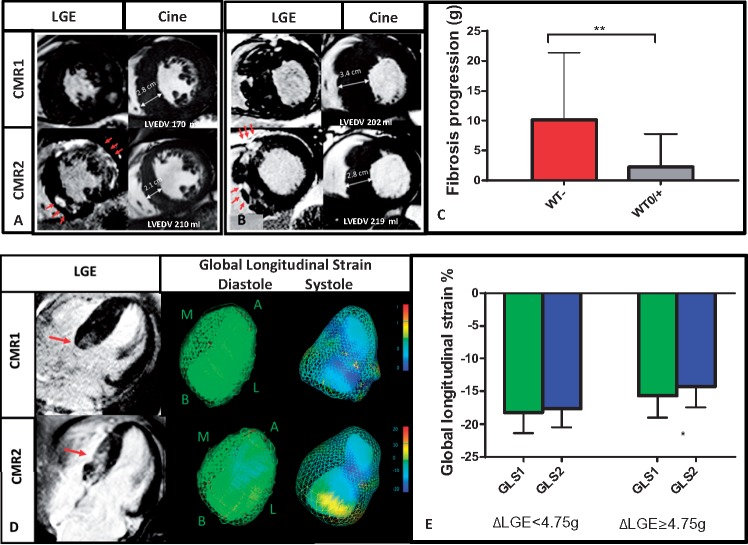
LGE/fibrosis progression (red arrow indicated LGE progression) results in a reduction in wall thickness (WT-) (*A*, *B*, *C*), increase in LV end-diastolic volume (*A*, *B*) and impairment in myocardial contractility (*D*, *E*) (WT0/- stable or increasing wall thickness; GLS, global longitudinal strain, **P < 0.01, error bars represent standard deviation, *P < 0.05).

Maximum end-diastolic LVWT did not differ significantly between the two CMR scans (19 ± 6 mm vs. 19 ± 5 mm, *P* = 0.79) (*Table 1*). Thirty-two (45%) had stable LVWT on follow-up, 24 (33%) had a modest increase (3.0 ± 1.6 mm) in LVWT, and 16 (22%) patients had a reduction of LVWT. Seven (10%) had a reduction of >3 mm. Interestingly, patients with regression of wall thickness (WT-) had a significantly higher extent of LGE increment (*Figure 3A–C*) vs. those with stable or increasing LVWT (WT0/+)- median LGE difference of 6.92 g (95% CI 2.72–10.40 g, *P* < 0.0001) between groups (*Figure 3C*). Two individuals with LVWT regression may have been reclassified to a lower risk group based on the assessment of traditional major SCD risk factors at CMR2 alone. Maximum LVWT at CMR1 correlated moderately with LGE progression (*r* = 0.36, *P* < 0.002). At CMR2, a weaker association was seen between LVWT and LGE progression (*r* = 0.28, *P* = 0.03). With regards to morphological variants, there were no differences in LGE increment between apical (*n* = 6) and non-apical HCM.

### Impact of substantial LGE progression on LV volumes and function

In the subgroup of patients with ΔLGE ≥ 4.75 g (*n* = 19), there was a significant increase in LVEDV from CMR1 to CMR2 (CMR1 161 ± 30 mL vs. CMR2 169 ± 37 mL, *P* = 0.04) (*Figure 3A* and *B*) with reduction in LVEF (CMR1 65 ± 7% vs. CMR2 62 ± 7%, *P* = 0.03) and GLS (−16 ± 3% vs. −15 ± 3%, *P* = 0.04) (*Figure 3D* and *E*). There was no difference in LV mass seen despite these changes.

### Relationship between genotype and LGE progression

Forty-five (63%) patients had sarcomeric mutations; three (4%) had mitochondrial mutation; three (4%) had a variant of uncertain significance in a sarcomeric gene; no pathogenic mutation was found in 21 (29%) patients (see Supplementary data online, *Table S1*). Nine patients were pre-hypertrophic (max LVWT ≤ 13 mm) sarcomeric mutation carriers (G+P−). On univariate analysis, genotype did not predict significant LGE progression.

None of the nine sarcomeric G+P− patients had progression of LGE ≥ 4.75 g over a CMR interval of 6 ± 3 years. However, LGE progression did occur in those with (G+P+) sarcomeric HCM (2.79 g IQR 1.12–7.39 g, *P* < 0.01) vs. G+P− patients (0.17 g, IQR 0.18–1.03 g) (see Supplementary data online, *Figure S2*). In patients with LVH (LVWT ≥ 15 mm), differences in LGE increments could also be seen between those with and without sarcomeric mutations. Mitochondrial mutation carriers had the highest median LGE increment of 23.16 g (IQR 16.84–45.78 g) (*P* < 0.01 for all comparisons) followed by sarcomeric mutation 2.79 g (IQR 1.12–7.39 g) and genotype negative patients 0.52 g (IQR −0.38 to 2.43, *P* = 0.01 for comparison between genotype negative and sarcomeric mutation) (see Supplementary data online, *Figure S2*).

### Impaired energetics and myocardial perfusion reserve are associated with LGE progression

**Figure 4:**
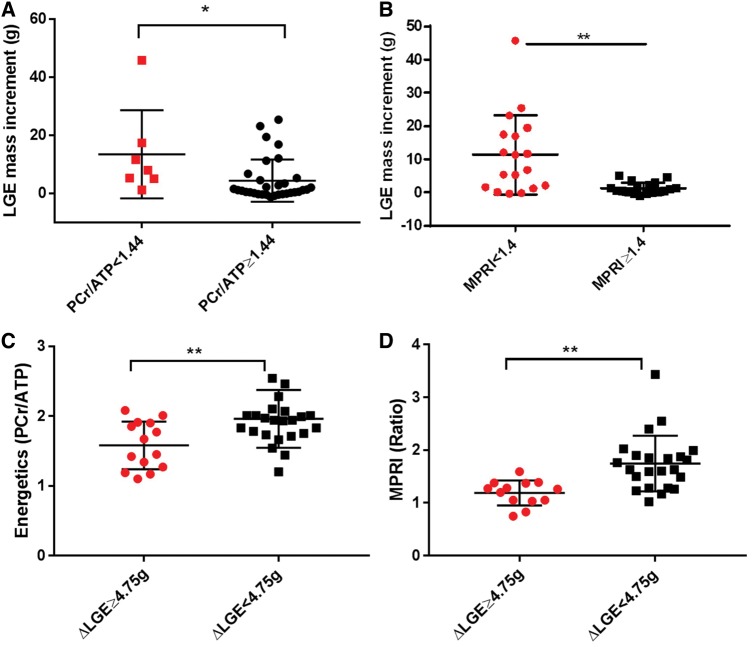
LGE mass increases from CMR1-CMR2 in HCM patients with (*A*) impaired myocardial energetics and (*B*) impaired myocardial perfusion reserve index at baseline CMR. (*C*) Myocardial energetics and (*D*) perfusion reserve index are impaired in those with substantial LGE progression. (MPRI, myocardial perfusion reserve index; PCr/ATP, phosphocreatine to adenosine triphosphate ratio; **P < 0.01, *P < 0.05, ΔLGE ≥ 4.75 g LGE progression of ≥4.75 g or substantial LGE increment, error bars represent standard deviation).

Myocardial energetics were assessed in 38 patients at CMR1. ΔLGE ≥ 4.75 g was seen in 14 patients. An impairment in energetics was defined as less than two standard deviations of previously reported healthy range (1.71 ± 0.35).^16^ In those with impaired energetics (PCr/ATP <1.44), there was a significantly higher LGE increment on follow-up compared with those with normal energetics (median increment 7.99 g IQR 5.01–17.41 g vs. 1.20 g IQR −0.05 to 25.39, *P* = 0.01) (*Figure 4A*). Additionally, patients with ΔLGE ≥ 4.75 g had reduced myocardial energetics at baseline compared with those with less progression (PCr/ATP 1.58 ± 0.34 vs. 1.96 ± 0.41, *P* = 0.006) (*Figure 4C*).

Adenosine first-pass perfusion imaging was performed in 35 patients at CMR1. Inducible perfusion abnormalities were seen in 25 patients and ΔLGE ≥ 4.75 g was seen in 13 patients. LGE progression commonly involved myocardial segments with inducible perfusion defects at baseline. Seven subjects developed *de novo* LGE in regions without inducible perfusion defects. Based on a previous study, an MPRI < 1.40 was considered suggestive of microvascular dysfunction.^25^^,^^26^ Patients with impaired MPRI on baseline CMR had a higher LGE increment on interval scans compared with normal MPRI (median 9 g IQR 1.47–17.91 g vs. 0.74 g IQR −0.08 to 2.37 g, *P* < 0.01) (*Figure 4B*). In patients with ΔLGE ≥ 4.75 g, MPRI was severely impaired on CMR1 compared with those with less progression (1.18 ± 0.23 vs. 1.74 ± 0.53, *P* = 0.001) (*Figure 4D*).

### Progression of fibrosis predicts clinical outcomes

In the final cohort of 72 patients, eight underwent primary prevention ICD implantation subsequent to CMR2. There were no deaths, aborted cardiac deaths or appropriate ICD shocks. Twenty-four had clinical events as previously defined. New onset ventricular tachycardia was detected in 13 patients. All cases of new onset NSVT were detected on 24 hour ECG monitor prior to the implantation of device. Progression of heart failure with optimization of therapy occurred in nine patients. Three hospital admissions occurred due to progression of heart failure symptoms and atrial fibrillation.

**Figure 5:**
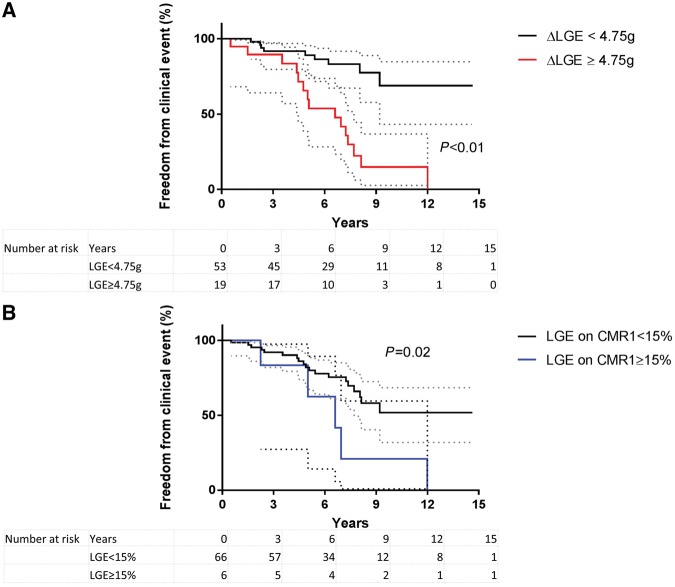
Kaplan–Meier curves depict the freedom from clinical events in HCM patients with LGE increment ≥4.75 g or less (*A*) and in those with LGE on CMR1 ≥ 15% of LV mass or less (*B*) (error bars represent 95% confidence intervals).

Amongst those with any LGE at baseline, 41% developed a clinical event during the follow-up period (CMR1 to end of study). In contrast, 79% of those with ΔLGE ≥ 4.75 g developed a clinical event on follow-up. In a univariate cox regression analysis, maximum LVWT on CMR1, initial LGE mass, ΔLGE ≥ 4.75 g were significant predictors of clinical outcomes (*Table 3*). On multivariable analysis, ΔLGE ≥ 4.75 g remained an independent predictor of outcome despite adjusting for age at outcome, maximum LVWT and LGE mass at CMR1 [hazard ratio (HR) 5.04, 95% CI 1.85–13.79; *P* = 0.002]. HCM patients with ΔLGE ≥ 4.75 g had a significantly lower freedom from clinical events compared with others (*Figure 5A*). Similarly, patients with baseline LGE of ≥15% of LV mass had a low freedom from clinical events but to a lesser extent than LGE progression (*Figure 5B*).

**Table 3:** Univariate and multivariable Cox regression analysis of predictors of clinical outcomes in HCM

	HR	95% CI	*P*-value
Univariate Cox			
Age at outcome	0.98	0.95–1.01	0.36
Gender	0.83	0.36–1.91	0.66
Maximum LVWT at CMR1	1.10	1.03–1.17	0.007
LV mass at CMR1	1.01	0.99–1.01	0.12
LGE mass at CMR1	1.04	1.01–1.07	0.002
LGE Progression ≥4.75 g	5.53	2.39–12.78	<0.001
Interval between CMR1-CMR2 (days)	1.00	0.99–1.00	0.39
LVEF CMR1	1.01	0.94–1.08	0.85
Apical vs. non-apical hypertrophy	2.21	0.63–7.33	0.22
Genotype^a^	1.94	0.66–5.69	0.23
Baseline SCD risk factors	0.51	0.21–1.25	0.14
Multivariable Cox			
Age at outcome	0.98	0.94–1.01	0.19
Maximum LVWT at CMR1	1.07	0.96–1.19	0.25
LGE mass at CMR1	0.98	0.94–1.03	0.52
LGE Progression ≥4.75 g	5.04	1.85–13.79	0.002

CI, confidence interval; CMR, cardiac magnetic resonance imaging; HR, hazard ratio; LVEF, left ventricular ejection fraction; LVWT, left ventricular wall thickness; SCD, sudden cardiac death.

a Sarcomeric and mitochondrial mutations vs. genotype negative.

## Discussion

Our study demonstrates that myocardial fibrosis, quantified by LGE, is progressive in a proportion of HCM patients, and clinically relevant LGE progression is characterised by adverse cardiac remodelling. We also provide novel insights into potential mechanisms of LGE progression including the relative contributions of underlying genetic mutations, impaired myocardial energetics and microvascular dysfunction, biological mechanisms believed to promote myocardial fibrosis, and hypertrophy in HCM.^4^^,^^13^^,^^27^ Although there were no deaths in this cohort, significant LGE progression in HCM strongly associated with the risk of escalating heart failure and arrhythmia symptomatology over time. These findings suggest that therapies with energy sparing, vascular protective or anti-fibrotic effects may be beneficial in preventing progression of heart failure and arrhythmic risk in HCM.

### Progression of myocardial fibrosis in HCM associates with adverse cardiac remodelling

LGE on CMR provides a robust and reproducible tool for the assessment of myocardial fibrosis in HCM.^4–6^ Previous small proof-of-principle studies examining LGE in HCM report an increase at intervals of 1.5–1.9 years.^10^^,^^11^ However, changes in LV function and adverse remodelling in HCM are a gradual process in HCM, likely to be missed on short interval scans.^28^ We, therefore, examined fibrosis progression over a longer interval. Consistent with others, we report a modest but significant increase in both LGE mass and relative LGE mass (proportion of LV mass). Although we observed a smaller amount of LGE progression compared with previous work (median LGE increment 6 g in Ref.^10^), we believe this likely reflects the lower baseline risk profile of our patients with the inclusion of G+P− patients in this cohort.

The extent of LGE at a single time point has previously shown to associate with LV dysfunction and incidence of heart failure.^8^ Interestingly, a recent study by Todiere *et al.*^10^ reported an increased burden of heart failure symptoms in those with higher LGE progression rate. Here, we show that HCM patients with clinically relevant LGE progression have evidence of adverse ventricular remodelling, including increased LV cavity size, reduced ejection fraction, and global longitudinal strain. These findings are unique to this study and possibly reflect the longer intervals between scans in this study. This may also explain the increased event rate in those with substantial LGE progression.

### Maximum LVWT can regress due to LGE progression

We did not see a significant increase in maximum wall thickness from CMR1 to CMR2. Instead, maximum thickness at CMR1 strongly associated with LGE progression suggesting that those with pre-existing hypertrophy tend to develop LGE progression. Importantly, we observed that while in some patients maximum wall thickness increased over time, others had regression associated with a significant rise in LGE burden. Indeed, previous studies have reported a link between LGE mass at a single time point and myocardial thinning on follow-up echocardiography.^29^^,^^30^ However, no study to date has systematically assessed the contribution of LGE progression to myocardial wall thinning. Importantly, in two of our subjects, this phenomenon lead to a reclassification of SCD risk to lower risk based on traditional risk factors at CMR2. Therefore, our data suggests that the assessment of maximum wall thickness without information on LGE burden may potentially underestimate the perceived SCD risk in individuals with HCM. These observations highlight a complex relationship between progression of LGE and degree of LV hypertrophy which may inform clinical protocols for follow-up surveillance.

### Genotype may influence LGE progression in the hypertrophied ventricle

We assessed the significance of underlying pathogenic mutations and LGE progression in HCM. Genotype did not associate with LGE progression, possibly due to the inclusion of G+P− patients in our cohort who did not develop hypertrophy or significant LGE progression. These findings are concordant with a previous study by Ho *et al*.,^31^ who showed that phenotype negative carriers lacked LGE despite evidence of increased collagen synthesis, due to a compensatory increase in collagen degradation.^31^ Interestingly, in those with sarcomeric mutation and overt hypertrophy, this dynamic equilibrium between collagen synthesis and degradation was lost resulting in a significantly higher LGE. Indeed, when we assessed sarcomeric mutation carriers with overt hypertrophy, patients had significantly higher LGE progression compared with those with a pre-hypertrophic phenotype (G+P–) (See Supplementary data online, *Figure S2*). Those with sarcomeric HCM were also found to have a higher burden of LGE progression than genotype negative patients. In a cross sectional study by Olivotto *et al*.,^32^ similar observations were made about the prevalence of LGE in sarcomeric mutation vs. genotype negative HCM. This suggests that factors arising either directly from the expression of sarcomeric mutations or due to modifier gene effects^33^^,^^34^ possibly promote both hypertrophy and fibrosis in sarcomeric HCM.^35^

Interestingly, patients with a metabolically deficient phenocopy of HCM—mitochondrial HCM showed the highest increase in LGE when compared with sarcomeric and genotype negative patients. Previous studies in transgenic mouse models of sarcomeric mutations also suggest that increased ATP utilization and altered calcium-dependent signalling may play a central role in disease progression in HCM.^27^ Our data provides further evidence that HCM characterized by ‘energy costly’ mutations are at greatest risk for fibrosis progression following the onset of LV hypertrophy. Here, we were unable to examine differences within genetic subgroups and given the limited scale of our study, further validation of our findings in a larger genotyped HCM cohort will most certainly be required.

### Impaired energetics and microvascular dysfunction underlie LGE progression in HCM

Impairment in myocardial energetics is important in the development of heart failure,^36^ but studies examining its long-term sequelae in HCM are lacking. Metabolic therapies that improve energetic deficits have been promising at improving functional capacity in HCM,^15^ though their role in preventing hypertrophy and fibrosis remains to be elucidated. Here, we found that HCM patients with impaired myocardial energetics at baseline have a higher burden of LGE progression at follow-up and conversely in those with substantial LGE progression, myocardial energetics were severely impaired. This suggests that impairment in myocardial energetics may contribute to the risk of fibrosis progression in HCM. Future studies evaluating the effects of metabolic therapies on LGE burden may provide valuable insights into the role of impaired energetics in disease progression.

The non-invasive assessment of microvascular dysfunction can predict adverse cardiac remodelling.^4^^,^^14^ Several cross-sectional studies report a strong association between microvascular dysfunction and LGE burden,^37^^,^^38^ but its role in promoting fibrosis is unclear. We observed that HCM patients with impaired myocardial perfusion reserve had more LGE progression. Importantly, in those with substantial LGE progression and adverse remodelling, myocardial perfusion reserve was severely compromised. It therefore follows that microvascular dysfunction may promote fibrosis progression which in turn causes adverse cardiac remodelling. The development of de novo regions of fibrosis suggests that factors other than microvascular dysfunction likely contribute to fibrosis progression in HCM including energetic impairment, pro-fibrotic signalling and inflammation.^13^^,^^31^^,^^39^

### Progression of fibrosis predicts composite clinical endpoints

We assessed if LGE progression could predict clinical events that could potentially alter clinical management. We found that ΔLGE ≥ 4.75 g was the strongest predictor of clinical sequelae with an age-adjusted HR of 5.02 (*P* = 0.02) on multivariable analysis despite adjusting for baseline LGE mass. These findings highlight the importance of longitudinal assessment of LGE as a dynamic pathological process, given its predictive capacity over and above a single measurement of LGE. Although LGE was seen in the majority of individuals at baseline, only 41% of them experienced a clinical event. On the other hand, 79% of those with LGE progression had a clinical event highlighting the common discordance between LGE prevalence and clinical outcome. Our study suggests that assessing the temporal profile of disease activity (LGE progression) rather than just the presence of LGE, a ‘fossil’ of disease activity, may help identify those at risk of clinical deterioration. This is further illustrated by the freedom from clinical event curves, which demonstrate that LGE progression was better than LGE extent ≥15% at CMR1 for discriminating those with an evolving disease from others with a stable clinical course.

### Study limitations

This is a single-centre study limited by relatively small sample size with low SCD risk due to the exclusion of those with ICD after CMR1. Despite our encouraging data, given the lack of hard clinical endpoints in our cohort, the prognostic value of LGE progression for major cardiovascular events requires further investigation—in this context, the recent large international multicentre Hypertrophic Cardiomyopathy Registry study (HCMR; *n* = 2764) could provide an ideal platform for repeat imaging of phenotype progression over time.^40^

The LGE technique used in this study detects mostly focal fibrosis. The accuracy of prevalence estimates of LGE progression may also be limited due to the small sample size. In this study, histological validation of LGE progression by endomyocardial biopsy was not feasible. However, previous studies of septal myomectomy and endomyocardial biopsies from HCM patients confirm a strong correlation between the extent of myocardial fibrosis detected on biopsy and LGE on CMR.^6^^,^^41^

We acknowledge that the use of different contrast agents at CMR1 and CMR2 and different field strengths for some patients on serial CMR assessments are potential limitations of this study, but there was no evidence that these factors affected the rate of LGE progression. The predictive value of LGE progression may be lower than the current estimates from the multivariable analysis due to over-fitting when applying the method prospectively.

Another limitation of this study is the grouping of patients with sarcomeric mutations, which may be an oversimplification, and larger longitudinal studies of LGE imaging in genotyped cohorts will be needed to assess potential differences among individual sarcomeric mutations.

## Conclusions

This study has demonstrated that clinically significant progression of myocardial fibrosis occurs in some adults (26%) with HCM over a 6-year period. Impairment of myocardial energetics and perfusion reserve may play a pathophysiological role. The detection of fibrosis progression on CMR advances our ability to identify patients at risk of developing adverse LV remodelling, heart failure progression and arrhythmia.

## Supplementary Material

Supplementary Material
