# Supplementary material for: Progression of myocardial fibrosis in hypertrophic cardiomyopathy: mechanisms and clinical implications
Source: Eur Heart J Cardiovasc Imaging. 2018 Oct 24;20(2):157–67. doi: 10.1093/ehjci/jey135 (PMC6343081; doi:10.1093/ehjci/jey135)
Supplement: Supplementary Material [file jey135_supplemental_material.docx]

# **Supplemental material**

**Traditional risk factors for sudden cardiac death**

Traditional risk factors were defined as the presence of : 1) severe LV hypertrophy (maximum LVWT>30 mm); 2) ventricular tachycardia (≥3 consecutive ventricular beats, ≥120 bpm) on 24-hour ambulatory (Holter) ECG monitoring; 3) family history of SCD ( first-degree relative, under 50 years of age); 4) unexplained syncope; 5) abnormal blood pressure response during exercise (under age of 50) (1).

**Genetic screening**

All patients underwent screening for 13 genes associated with HCM (MYBPC3: myosin binding protein C; MYH7: myosin heavy chain; TNNI3: cardiac troponin I; TNNT2: cardiac troponin T; MYL2: regulatory myosin light chain; MYL3: essential myosin light chain; TPM1: alpha tropomyosin; ACTC1: cardiac actin; CSRP3: muscle LIM protein; PRKAG2: AMPK γ2; PLN: phospholamban; GLA: alpha galactosidase; FHL1: four and a half LIM domains 1) including a screen (blood test) for mitochondrial mutations if no sarcomeric mutations were identified.

**Exclusion of coronary disease in final cohort**

Of the 72 patients undergoing serial CMR, coronary angiography was performed in those patients (n=15) with a clinical indication such as symptoms of angina or positive exercise stress test. No significant coronary disease could be detected in any of the fifteen. The remaining patients with negative stress tests had a low cumulative Framingham Risk for coronary disease of <10% with no evidence of myocardial infarction on LGE imaging.

**Methods**

**Cardiac magnetic resonance cine imaging**

Cardiac volumes were acquired using steady state free precession (SSFP) imaging. Scan parameters were typically: voxel size 2.0x2.0x8.0mm, FOV=380x380mm, TR/TE 39.6/1.12ms, flip angle 55°, matrix 192x192, GRAPPA=3, 24 reference lines, segments=15, concatenations=1. Pilot images were initially acquired and used to plan and acquire horizontal long axis (HLA), vertical long axis (VLA), left ventricular outflow tract (LVOT) long axis and short axis stack images. LV short axis epicardial and endocardial borders were manually contoured at end diastole and end systole. LV end systolic (ESV) and end diastolic (EDV) volumes were used to calculate stroke volume (SV) as SV = EDV-ESV. Ejection fraction (EF) and cardiac output (CO) were calculated as EF = SV/EDV and CO=SV x HR, respectively. LV mass was calculated by subtracting the endocardial volume from the epicardial volume, based on prior knowledge of myocardial specific gravity (1.05 g/cm^3^).

**CMR field strength**

The majority (78%) of paired scans were undertaken at the same field strength. All patients undergoing perfusion and energetics assessment (n=38) had serial CMR at 3 Tesla (T). 18 patients had serial CMR at 1.5T. Sixteen (22%) patients had LGE assessment at different field strengths (1.5T or 3T). All 3 Tesla (3T) scans were undertaken on 3 Tesla (3T), Tim Trio MR System, (Siemens, Erlangen, Germany). All 1.5T scans were undertaken either on Sonata or Avanto Fit, Siemens (Erlangen, Germany).

**Late gadolinium enhancement (LGE) imaging**:

LGE imaging was acquired using a T1-weighted phase-sensitive inversion recovery sequence. Scan parameters were typically: voxel size 2.0 x 1.5 x 8.0 mm, matrix 144x256, field-of view=380x285mm, TR/TE=800.20/3.36ms, flip angle 25^o^, GRAPPA=2, 24 reference lines, segments=25, phases=1, concatenations=1, measurements=1, bandwidth=130Hz/Px.

**^31^P magnetic resonance spectroscopy**

A 3-dimensional acquisition-weighted chemical shift imaging technique (3D UTE-CSI) was used with an acquisition matrix of 16 x 8 x 8 over field of view of 240 x 240 x 200 mm^3^ with 10 averages at the centre of k-space (2). The sequence used the ultrashort echo time (UTE) approach to minimize T_2_ effects and first-order phase artefacts. The total acquisition time was ~9 min. An optimized radiofrequency pulse centred between the γ- and α-ATP resonance frequencies was used to ensure uniform excitation of all spectral peaks. Five Nuclear Overhauser Effect (NOE) pulses (2.5 ms, 222.2 V separated by 80.5 ms) were used to increase signal to noise. Three 25-mm-thick saturation bands were used to minimise signal contamination in the heart, 2 placed over chest wall muscle and 1 placed over liver. The chemical shift imaging grid was placed with a central voxel in the mid-ventricular septum and rotated to maximize coverage of the septal myocardium.

**CMR Image Post-Processing**

Cine images:

Analysis of left ventricular ejection fraction (LVEF) was performed using the cmr42 software (Circle Cardiovascular Imaging Inc., Calgary, Canada). LV short-axis epicardial and endocardial borders were manually contoured at end-diastole and end-systole, in accordance to the SCMR guidelines on standardized image post-processing of CMR images (3)*.* Papillary muscles were not included in the LV blood pool for LV volume contours. LV end-systolic (LVESV) and end-diastolic (LVEDV) volumes were used to calculate stroke volume (SV) and LVEF [LVEF = SV/EDV]. LV myocardial mass was calculated by subtracting the endocardial volume from the epicardial volume, based on prior knowledge of myocardial specific gravity (1.05 g/cm^3^). Left atrial diameter was measured in the LV outflow tract (3-chamber) view.

*Myocardial perfusion reserve analysis:*

For analysis of myocardial perfusion, signal intensity (SI) over time curves was generated by tracing endocardial and epicardial contours (cmr42) after correction for displacement during breathing. A region of interest was drawn in the LV blood pool to obtain an arterial input function. Post-adenosine rest and stress myocardial perfusion up slopes were calculated using a five-point linear fit model of SI vs. time and normalized to the LV blood pool upslope. Myocardial perfusion reserve index (MPRI), defined as the ratio of stress to rest normalized myocardial perfusion upslope, was derived for 18 segments and averaged per patient (4).

*^31^P Spectroscopy analysis:*

The spectrum from a mid-ventricular septal voxel was fitted using a custom implementation of AMARES (the advanced method for accurate, robust, and efficient spectral fitting) in the “OXSA” semi-automated spectroscopy post-processing pipeline (5). Fitting used prior knowledge specifying 11 Lorentzian peaks (α,β,γ-ATP multiplet components, PCr, PDE, and 2x2,3-DPG) and fixed amplitude ratios and scalar couplings for the multiplets. The fitted amplitudes were then corrected for blood contamination by subtracting 30% of the average of the two 2,3-DPG signals from each of the ATP amplitudes. The remaining PCr and ATP signals were corrected for the effects of partial saturation using the flip angle at the centre of the voxel, assuming no motion effects and with literature T_1_ values.

**Visual assessment and semi-quantitative analysis of LGE**

Visual assessment of LGE progression was also performed to ensure that LGE progression ≥4.75g using a semi quantitative method was detectable by an expert clinician (MM) on blinded analysis. Level of agreement was assessed to be high with Cohens kappa 0.90 (95% CI 0.79-1.00), p<0.01. Inter-observer intra-class correlation coefficient (two-way mixed effect) for (n=15) was excellent at 0.89 (95% CI 0.80-0.95, p<0.01), intra-observer ICC (one-way random effect) was also found to be high 0.97 (95% CI 0.95-0.99, p<0.01).

**LGE increment threshold**

A receiver operator curve analysis was undertaken to estimate a clinically meaningful LGE threshold. We found that substantial LGE increment of 4.75g (ΔLGE≥4.75g) had a good specificity of 92% (95% CI 85%-97%) and reasonable sensitivity of 63% (95% CI 53%-72%) with an AUC of 0.74 (95% CI 0.60-0.88, p=0.001) for discriminating stable patients from those likely to have a clinical event (Supplementary figure 1).

**LVOT Obstruction and LGE progression**

In this study, there were 11 patients with LV outflow tract obstruction at rest or during Valsalva (>30mm Hg), and three demonstrated evidence of substantial LGE progression. There was no significant difference in LGE increment between those with and without LVOT obstruction (p=0.12). Out of those 11 patients, two had clinical events, one had a progression of NYHA class with a restrictive physiology on echocardiogram, the other had new onset ventricular tachycardia and atrial fibrillation. The presence of LVOT obstruction did not significantly associate with LGE progression or clinical events on univariate analysis. However, numbers are small and the present study lacks the power to robustly test such associations.

**Data**

**Table 1. Genotype and patterns of hypertrophy of HCM subjects enrolled in study**

| Genotype |  |
| --- | --- |
| MYH7, %(n) | 26(19) |
| MYBPC3, %(n) | 32(23) |
| ACTC1, %(n) | 1(1) |
| TNNI3, %(n) | 1(1) |
| MYL, %(n) | 1(1) |
| Mitochondrial, %(n) | 4(3) |
| VUS (MYBPC3, MYH7) | 4(3) |
| Gene negative, %(n) | 29(21) |
| Pattern of hypertrophy (6) |  |
| Normal, %(n) | 13(9) |
| Septal, %(n) | 64(46) |
| Reverse septal, %(n) | 3(2) |
| Mid-ventricular, %(n) | 0(0) |
| Apical, %(n) | 9(6) |
| Concentric, %(n) | 12(9) |
| NSVT, non-sustained ventricular tachycardia; SCD, sudden cardiac death; HCM; Hypertrophic cardiomyopathy; MYH7, beta-myosin heavy chain; MYBPC3, myosin-binding protein C, TNN1, Troponin, ACTC1 Alpha cardiac actin; MYL myosin light chain | |

**Table 2. Baseline characteristics of patients with HCM and progression of LGE ≥4.75g**

|  | Progression of fibrosis  (n=19) | | No progression  (n=53) |  |
| --- | --- | --- | --- | --- |
| Age (years) | | 46 ± 12 | 45 ± 12 |  |
| Male%, (n) | | 12(63) | 37(70) |  |
| Body mass index (kg/m^2)^ | | 29±5 | 27±5 |  |
| Hypertension, (n) | | 1 | 6 |  |
| Diabetes, (n) | | 2 | 0 |  |
| Smoker, (n) | | 1 | 4 |  |
| Family history of SCD, (n) | | 2 | 17 |  |
| Unexplained syncope, (n) | | 1 | 2 |  |
| NSVT on Holter monitor(n) | | 2 | 5 |  |
| Abnormal exercise BP response, (n) | | 0 | 1 |  |
| Maximum LV wall thickness ≥30 mm, (n) | | 22±5 | 17±5 |  |
| Presence of LVOT gradient (n) | | 3 | 8 |  |
| (0/1/2/3 risk factors), (n) | | 14,4,1,0 | 30,20,3,0 |  |
| ESC 5-yr estimated SCD risk, % | | 3.0±2.2 | 2.1±0.9 |  |
| Medications | |  |  |  |
| β-Blockers, (n) | | 8 | 28 |  |
| Calcium channel blockers, (n) | | 3 | 3 |  |
| Disopyramide, (n) | | 3 | 1 |  |
| ARB/ACEI , (n) | | 3 | 7 |  |
| Diuretics, (n) | | 2 | 2 |  |
| Warfarin ,(n) | | 2 | 2 |  |
| Aspirin, (n) | | 4 | 14 |  |
| CMR findings | |  |  |  |
| LVEF, % | | 65±7 | 68±6 |  |
| LVEDV, ml | | 161±30 | 149±29 |  |
| LVESV, ml | | 56±17 | 49±14* |  |
| Stroke volume, ml | | 103±20 | 101±20 |  |
| LA diameter (LVOT/3ch view) | | 39±7 | 36±5 |  |
| LV Mass, g | | 178±60 | 135±46* |  |
| LV Mass/m^2^ | | 89±30 | 70±21* |  |
| Max wall thickness, mm | | 23±5 | 17±5* |  |
| Data are mean ± standard deviation. | | | | |
| LV Left ventricular; LA left atrial; EDV end-diastolic volume; ESV end-systolic volume; EF ejection fraction; HCM hypertrophic cardiomyopathy; LGE, late gadolinium enhancement (5 SD), ACEI angiotensin converting enzyme inhibitor; BP blood pressure; NSVT Non sustained ventricular tachycardia; SCD Sudden cardiac death; LVOT left ventricular outflow tract, *comparison significantly different, p<0.05 | | | | |

**
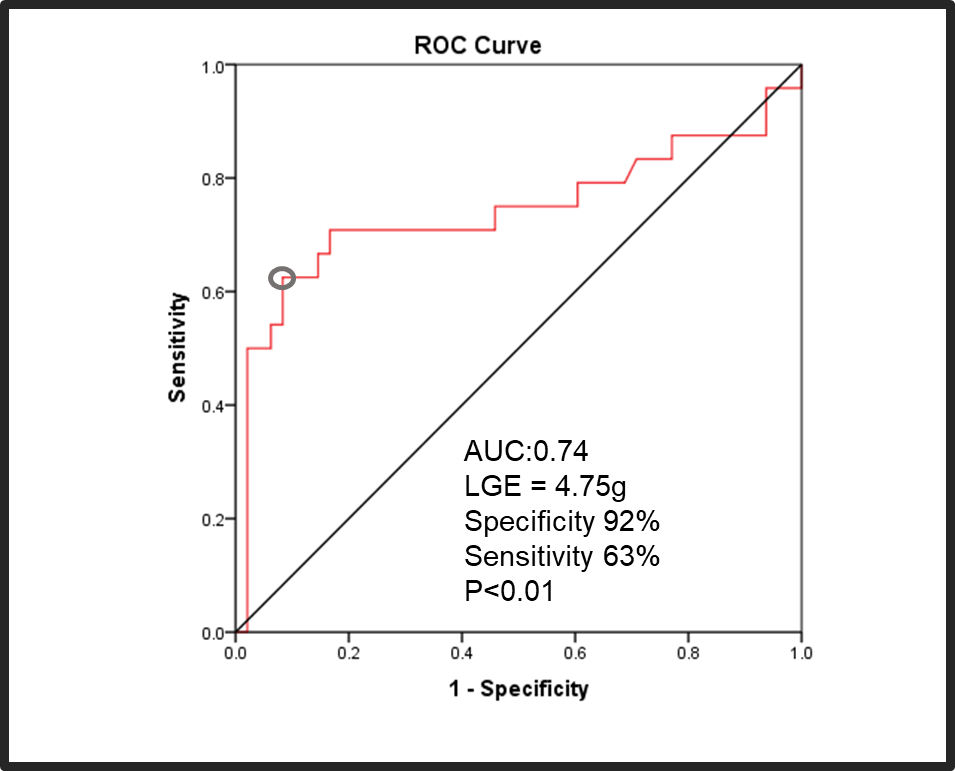
**

**Supplemental Figure 1 HCM patients who develop clinical events had a higher degree of LGE progression on serial CMR compared to stable patients B. Receiver Operator Curve depicts that an LGE increment threshold of 4.75 g (Youden index) has good specificity of 92% and reasonable sensitivity of 63% to detect clinical events.**

**Supplemental Figure 2. Comparison of LGE increment between those with and without sarcomeric mutations in HCM.**

**References for Supplemental Section**

1. Gersh BJ, Maron BJ, Bonow RO, Dearani JA, Fifer MA, Link MS, et al. 2011 ACCF/AHA guideline for the diagnosis and treatment of hypertrophic cardiomyopathy: executive summary: a report of the American College of Cardiology Foundation/American Heart Association Task Force on Practice Guidelines. J Am Coll Cardiol. 2011;58(25):2703-38.

2. Tyler DJ, Robson MD, Henkelman RM, Young IR, Bydder GM. Magnetic resonance imaging with ultrashort TE (UTE) PULSE sequences: technical considerations. J Magn Reson Imaging. 2007;25(2):279-89.

3. Schulz-Menger J, Bluemke DA, Bremerich J, Flamm SD, Fogel MA, Friedrich MG, et al. Standardized image interpretation and post processing in cardiovascular magnetic resonance: Society for Cardiovascular Magnetic Resonance (SCMR) Board of Trustees Task Force on Standardized Post Processing. Journal of Cardiovascular Magnetic Resonance. 2013;15(1):1-19.

4. Nagel E, Klein C, Paetsch I, Hettwer S, Schnackenburg B, Wegscheider K, et al. Magnetic resonance perfusion measurements for the noninvasive detection of coronary artery disease. Circulation. 2003;108(4):432-7.

5. Purvis LAB, Clarke WT, Biasiolli L, Valkovic L, Robson MD, Rodgers CT. OXSA: An open-source magnetic resonance spectroscopy analysis toolbox in MATLAB. PLoS One. 2017;12(9):e0185356.

6. Noureldin RA, Liu S, Nacif MS, Judge DP, Halushka MK, Abraham TP, et al. The diagnosis of hypertrophic cardiomyopathy by cardiovascular magnetic resonance. J Cardiovasc Magn Reson. 2012;14:17.
